# Assessing the patterns and drivers of shape complexity in the amblypygid pedipalp

**DOI:** 10.1002/ece3.7882

**Published:** 2021-07-14

**Authors:** Callum McLean, Russell Garwood, Charlotte Brassey

**Affiliations:** ^1^ Department of Natural Sciences Manchester Metropolitan University Manchester UK; ^2^ School of Earth and Environmental Sciences University of Manchester Manchester UK; ^3^ Earth Sciences Department Natural History Museum London UK

**Keywords:** Amblypygid, elliptical fourier analysis, pedipalp, shape complexity

## Abstract

Amblypygi is an arachnid order possessing a unique pair of spined pedipalps: appendages that perform in prey capture, courtship, and contest. Pedipalp length, hypothesized to be under sexual selection, varies markedly across amblypygid species, and pedipalp spination, thought to reflect selection for function in prey capture, also differs interspecifically. Differences in pedipalp shape between species may indicate that the relative strength of selection for prey capture and sexual selection vary across the group. However, interspecific differences in pedipalp shape have not been quantified, due to difficulties in identifying homologous features. For the first time, we quantify trends in amblypygid pedipalp shape complexity. We use elliptical Fourier analysis to quantify 2D complexity in pedipalp outlines across eleven species and six genera. We find that complexity significantly decreases as pedipalp length increases. This appears to be driven by relative spine length, suggesting that a trade‐off exists between pedipalp length and spination. Furthermore, significant female‐biased sexual dimorphism in shape complexity is present in the tibial segment of the amblypygid pedipalp. Our results provide novel insights into the drivers of amblypygid pedipalp evolution and suggest that a functional trade‐off between performance in prey capture and other functions under sexual selection exist in this enigmatic structure.

## Graphical abstract

Amblypygids are an arachnid order possessing a unique pair of spined pedipalps: appendages that perform in prey capture, courtship, and contest. We quantify trends in amblypygid pedipalp shape complexity using elliptical Fourier analysis applied to pedipalp outlines. Our results provide novel insights into the drivers of amblypygid pedipalp evolution and suggest that a functional trade‐off between performance in prey capture and other functions under sexual selection exist in this enigmatic structure.

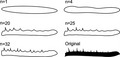

## 1 INTRODUCTION

Amblypygids are a group of predatory arachnids bearing a unique pair of raptorial pedipalps. The order comprises ca. 220 extant species (McArthur et al., 2018). The raptorial appendages in amblypygids are homologous to the claw‐bearing limbs of scorpions and thelyphonids that are used for prey capture in those groups, and to the limb bearing the palpal bulb in male spiders, which is used as a means of transferring sperm during mating. In common with other arachnids, the amblypygid pedipalp performs multiple functions, most notably prey capture (Santer & Hebets, 2011; Weygoldt, 2000). However, recent work has also highlighted the importance of the pedipalps in display (Chapin & Reed‐Guy, 2017), courtship (Chapin & Hebets, 2016; Weygoldt, 2000) and in the build‐up to contest (Chapin, 2015; Fowler‐Finn & Hebets, 2006; Santer & Hebets, 2011). Indeed, the majority of territorial disputes are also decided via display‐based contest (Chapin & Reed‐Guy, 2017). Pedipalps are also used in physical contest (Alexander, 1962; Weygoldt, 2000), and for drinking and grooming (Shultz, 1999; Weygoldt, 2000).

Amblypygid pedipalp morphology is markedly different from other arachnid orders, with palpal tibiae and femora taking an elongate and spinose form. Pedipalps also display a high level of interspecific morphological variation (Weygoldt, 2000). Across the group, they vary greatly in both absolute length, and in length relative to body size. For example, adult members of the genera *Sarax* and *Charinus* are characterized by pedipalps with a combined femur and tibia length equal to approximately one body length (Jocque & Giupponi, 2012; Rahmadi et al., 2010), while members of *Euphrynichus* and *Phrynichus* possess palps with combined femur and tibia lengths four times that of their body length (Simon & Fage, 1936; Weygoldt, 2000). Shape is also known to vary considerably across the group, with the position, number, relative length, and curvature of the pedipalp spines differing markedly among species (Weygoldt, 2000). However, shape diversity in the amblypygid pedipalp has been poorly quantified: Most information on shape diversity is limited to qualitative data or simple ratios (McArthur et al., 2018; McLean et al., 2018; Weygoldt, 2000).

This historic focus on gross pedipalp size has likely restricted our understanding of the functional ecology of these unusual appendages. Amblypygid pedipalps, like those of many other arachnid orders, are sexually dimorphic (McArthur et al., 2018; McLean et al., 2018; Weygoldt, 2000). Yet most studies of sexual dimorphism in amblypygids have focused solely on dimorphism in pedipalp length, with the limited work previously directed at shape dimorphism being qualitative (McLean et al., 2018). However, modern morphometric techniques can be used to elucidate previously undocumented shape dimorphism. For the first time, McLean et al. (2020) quantified shape variation within the pedipalps of a single amblypygid species using 2D geometric morphometric (GMM) analysis and identified significant sexual dimorphism within the spines, and relative width of the central shaft. That study discussed the potential importance of display‐based conflict and divergent reproductive roles in driving pedipalp sexual dimorphism (McLean et al., 2020) and highlighted the need to quantify shape variation across the order more broadly.

Indeed, studies using modern statistical shape analysis techniques have led to a clearer understanding of shape evolution in invertebrate weapons, and the link between shape and function. For example, geometric morphometrics (GMM) analysis of the harvestman species Forsteropsalis pureora has revealed male trimorphism in the chelicerae (Powell et al., 2020). Morphs with differing cheliceral shape engaged in different behavior in male–male contest, suggesting a link between chelicerae shape and function in contest (Powell et al., 2020). Similar studies in beetles have used a mixture of GMM and traditional morphometric methods to investigate links between mandible shape and function in intrasexual contest (Goczał et al., 2019; Matsumoto & Knell, 2017; Mills et al., 2016; Romiti et al., 2017). In particular, authors have noted that changes in mandible curvature and dentition in Coleoptera correlate with increasing mandible size (Matsumoto & Knell, 2017; Romiti et al., 2017). As mandible length is a strong predictor of the number of fights won, these shape changes are thought to play a key role in contest (Romiti et al., 2017).

However, while many of such studies have increased our understanding of the importance of shape evolution and the function of invertebrate weapons, interspecific studies using statistical shape methods are rare. That such an interspecific study of amblypygid pedipalp morphology has not been previously undertaken is perhaps surprising. Pedipalp morphology has long been considered diagnostic at a species level (Weygoldt, 2000), and pedipalp‐based characters have been used extensively in the construction of morphological amblypygid phylogenies (Garwood et al., 2017; Prendini et al., 2005; Weygoldt, 1996). Furthermore, documented amblypygid behavior is complex and varies markedly across the order, incorporating diverse social dynamics, varying degrees of territorially, differing mating strategies, and disparate feeding behaviors (Chapin & Reed‐Guy, 2017). Such factors have the potential to heavily influence the evolution of pedipalp shape.

This lack of multispecies comparisons may in part be attributed to difficulties in defining pedipalp spine homology (Weygoldt, 1998, 1999). Common shape analysis methods such as GMM require the manual/semi automatic placement of “landmarks” upon homologous features that are readily identifiable across all individuals in a sample. Within a given amblypygid taxon, spination is remarkably consistent in terms of gross pattern, and McLean et al. (2020) therefore proceeded with manual landmarking on the most prominent four spines. Between species however, spination is highly irregular, homology is difficult to determine by visual inspection, and the toolkit of evolutionary developmental biology has yet to be brought to bear on the genetic basis of pedipalp morphology. Thus, any comparison of shape across the amblypygid pedipalp must avoid assumptions of spine homology and employ non–landmark‐based methods.

Recently, developed tools for quantifying shape complexity offer a potential solution, as they do not rely on the placement of homologous landmarks. Shape complexity is distinct from the metrics of shape variation calculated by GMM and can broadly be defined as the number of “simple shapes” required to create a more complex shape, and the selfsimilarity of those composite parts (Chambers et al., 2018; Gardiner et al., 2018). Recent research has deployed shape complexity metrics to investigate a number of biological systems. For instance, tooth complexity has been related to dietary differences in primates and reptiles (Melstrom, 2017; Prufrock et al., 2016). Shape complexity has also been used on a number of invertebrate systems including the genitalia of water striders (Rowe & Arnqvist, 2012) and Drosophila wings (Ray et al., 2016).

Here, we apply the marker‐less method elliptical Fourier analysis, which has previously been to investigate shape variation in spiders and harvestmen (Bond & Beamer, 2006; Sharma et al., 2017). In this study, we quantify shape complexity of the amblypygid pedipalp across 11 species in 6 genera. For the first time, this facilitates a quantitative comparison of interspecific pedipalp shape across this important order.

### 1.1 Aims and hypotheses

We aim to establish a methodology for quantifying amblypygid pedipalp complexity in the absence of homologous landmarks and to characterize the patterns in gross shape complexity across the group. Our goal is to understand its evolutionary drivers. Specifically, we hypothesize:

Hypothesis 1 Given the pedipalps' previous roles as taxonomic characters, we expect that species can be statistically differentiated from one another on the basis of pedipalp outline shape.

Hypothesis 2 Across species, shape complexity will decrease with relative pedipalp length (normalized to body size), trading off shape complexity for segment elongation. Previous GMM analysis within *Damon variegatus* found relative spine length to decrease with increasing pedipalp length (McLean et al., 2020), yet the degree to which this intraspecific pattern in static allometry holds true across the order is unclear.

Hypothesis 3 Females will possess higher palpal complexity than males. This is likewise informed by McLean et al. (2020), in which relative spine length was found to be significantly higher in female *Damon variegatus* than males.

## 2 METHODS

### 2.1 Data collection and preparation

We used a sample of individuals from 11 species to investigate shape complexity in the amblypygid pedipalp (*Phrynichus exophthalmus, Damon diadema, Damon medius, Heterophrynus longicornis, Phrynus whitei, Acanthophrynus coronatus, Phrynus barbadensis, Paraphrynus viridiceps, Phrynus longipes, Paraphrynus aztecus, Paraphrynus williamsi*). This incorporated 82 individuals in the tibia analysis and 77 in the femur analysis. Disparity in the number of specimens between analyses resulted from the exclusion of individuals with damaged pedipalp segments. Species were chosen to represent a diversity of pedipalp lengths and morphotypes, and cover a large taxonomic range, spanning two of the five amblypygid families and encompassing six out of seventeen amblypygid genera. Specimens were identified by taxonomists based on morphology. The species chosen also span a wide geographic range, originating from South and Central America, Africa, and Asia. Multiple replicates of males and females were collected for each species to facilitate comparisons between the sexes (see Supplementary Material S1). All specimens were held in spirit at the Royal Central African Museum (Tervuren, Belgium), the Natural History Museum (London, England), The American Museum of Natural History (New York, USA), or the Natural History Museum in Vienna, Austria.

Photographs were taken with a Canon EOS D750 attached to a copy stand to ensure the camera remained perpendicular to the specimens. Photographs of smaller specimens were taken with a 60 mm macro lens, larger specimens were photographed with 18–55 mm telescopic lens. Pedipalps were not detached from specimens, but were orientated in a standard position parallel to the camera lens. The pedipalps occupied as much of the camera field of view as possible in order to minimize differences in relative image resolution between species. Binarised outlines were subsequently obtained from photographs by manually tracing the contour of the dorsal surface of the femur and tibia segments in Inkscape (Inkscape Project, 2020). Outlines were then converted to JPEG images in preparation for morphometric analysis.

Linear metrics of pedipalp length and overall body length (taken as a proxy for overall body size) were taken using digital calipers with a measurement precision of 0.01 mm. Relative pedipalp length was defined as pedipalp tibia length divided by body length. Blind repeated measures were taken on a set of *Damon variegatus* of varying size, and the mean measurement error was 2.80% (*SD*—1.46%, 10 specimens). All specimens were sexed by lifting the genital operculum in order to determine the presence/absence of the spermatophore organ. Any individual listed as juvenile or immature was excluded from the analysis. Wherever possible, linear measurements and photographs were collected from the right pedipalp. However, in instances where the right pedipalp was damaged, the left pedipalp was mirrored and used instead (*n* = 12).

### 2.2 Morphometric analyses

Due to the current lack of data regarding pedipalp spine homology across amblypygid species, here we set out to quantify geometric complexity using non–landmark‐based morphometric methods. We thus use elliptical Fourier analysis on binarized outlines of amblypygid pedipalps. All methods of measuring 2D shape complexity were implemented using the R package “Momocs” (Bonhomme et al., 2014).

### 2.3 Elliptical Fourier analysis

Pedipalp outlines were analyzed using elliptical Fourier analysis (EFA). EFA uses the principle of conventional Fourier analysis, which states that the *xy* coordinates of a circle or simple ellipse can be described by a set of sine and cosine waves referred to as harmonics. During the analysis, complex shapes are described by a series of “epicycles,” where a point moves around an ellipse of harmonic n which in turn moves around the perimeter of a larger ellipse of harmonic n‐1 and so on, creating a series of moving ellipses which “draw” out the prospective shape (Caple et al., 2017). A shape traced by a number of moving ellipses can thus be described by a series of sine and cosine waves, which are themselves described by a series of harmonic coefficients. Each harmonic is essentially layered on top of each other, with each harmonic describing a further level of shape complexity (Caple et al., 2017). As further harmonics are added to the model, the harmonics produce an outline that is closer to the original shape and captures more of its complexity, with a hypothetical harmonic model containing infinite harmonics drawing out an exact replica of the original shape (Caple et al., 2017).

Binarised pedipalp images were imported as JPEGs into R and were subsequently converted into a series of 10,000 equally spaced *xy* coordinates representing each outline. Outlines were rotated such that their principal axes aligned with the global x‐axis and then centered with their centroid at the global origin (0,0). The effect of scale was also removed from the analysis by normalizing all outlines to centroid size, the square root of the sum of squared distances of all the outline points of an object from their centroid or central point. A Procrustes fit was also required to align shapes. This method of alignment is often employed in studies that use elliptical Fourier analysis to quantify shapes with few major protrusions such as orca fins, human crania, and posterior lobes of Drosophila (Emmons et al., 2019; Friess & Baylac, 2003; Takahara & Takahashi, 2015). Despite a workflow intended to avoid alignment via landmarks (due to the problems of homology), pedipalps with few large spines or protrusions aligned solely through principal axes often appeared flipped about their long axis relative to other pedipalps, this is phenomenon has been previously documented for shape with high aspect ratio shapes (Bonhomme et al., 2014). To overcome this issue, three landmarks were assigned to the femoral segments, and four to the tibia on the proximal and distal end points of the pedipalp. These landmark points did not include pedipalp spines and thus did not encounter the issue of homology (see Supplementary Material S3 for location of landmarks). The Procrustes fit between these numbered landmarks prevented pedipalps from flipping.

**FIGURE 1:**
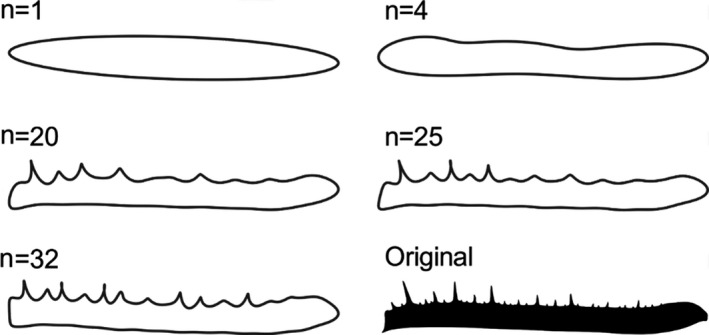
Outlines created by fitting Fourier models of varying harmonic order in the femur segment of *Damon diadema*

EFA was carried out using 32 harmonics, as this number was found to describe 99.9% of shape complexity of the original outline across the sample, in both tibia and femur segments. From the resulting harmonic fits, we calculated shape complexity. This was achieved by comparing perimeters, between EFA shapes of contrasting harmonics. Comparing boundary measures, such as perimeter, is a tenet of many methods of estimating shape complexity (Chambers et al., 2018). The first metric calculated is a low perimeter ratio that here we term “gross complexity.” This is calculated by dividing the perimeter of the outline created by fitting a complex 20th harmonic model, by the perimeter of a simple 4th harmonic model (see Figure 1), such that high values indicate high levels of gross complexity. Comparing 4th and 20th harmonic fit models has previously been used to quantify overall shape complexity in studies of other biological structures (Rowe & Arnqvist, 2012).

### 2.4 Data analysis

All data analysis was carried out in R. A principal component analysis was conducted on all harmonic coefficients derived from the Fourier analysis using Momocs (Bonhomme et al., 2014), in order to characterize intraspecific and interspecific variation in pedipalp shape complexity. The resulting PC scores were subject to clustering analysis in order to determine the degree to which species may be correctly identified on the basis of Fourier outlines. PC scores were scaled to have the same mean and standard deviation before being fed into the clustering algorithm. An unsupervised k‐means clustering was applied; k‐means was instructed to split the dataset into 11 clusters, the same as the number of species, in order to determine whether clustering would split down species lines. A pairwise MANOVA was also conducted in “Momocs” (Bonhomme et al., 2014).

To test the relationship between pedipalp shape complexity and length, species‐average values of “gross complexity” were calculated. Due to uncertainty in amblypygid phylogeny, ordinary least squares regression was then carried out between measures of complexity and pedipalp length for each segment individually. Pedipalp tibia length is considered a standard metric of pedipalp length and is used in multiple studies (McArthur et al., 2018; Prendini et al., 2005; Weygoldt, 2000). As such, this was the measure of pedipalp length used herein. A Shapiro–Wilk test confirmed that shape complexity metrics for both segments followed a normal distribution.

Tests for sexual dimorphism in shape complexity were carried out using a nested ANOVA between measures of complexity and sex, with species included as a random effect. Shapiro–Wilk tests confirmed shape complexity in both metrics was normally distributed within species, and species standard deviations were very similar in all species.

A paired Wilcoxon rank‐sum test was used to investigate differences in shape complexity between segments, as data were non‐normally distributed. Specimens that did not appear in both the tibia and femur dataset were excluded from this analysis, resulting in a total sample size of 75.

## 3 RESULTS

### 3.1 Evaluation of shape complexity metrics

Our first task was to assess what component of shape was driving increased complexity scores across the dataset. We define “complexity” as the difference between the 4th and 20th harmonic model. On average across all taxa, the 4th harmonic model describes 87.9% of the complexity of the original femur shape and 85.5% in the tibia, while the 20th harmonic model describes around 99.7% of the complexity of the original segment shape both segments. Thus, gross complexity comprises approximately 14% of the total shape complexity.

**FIGURE 2:**
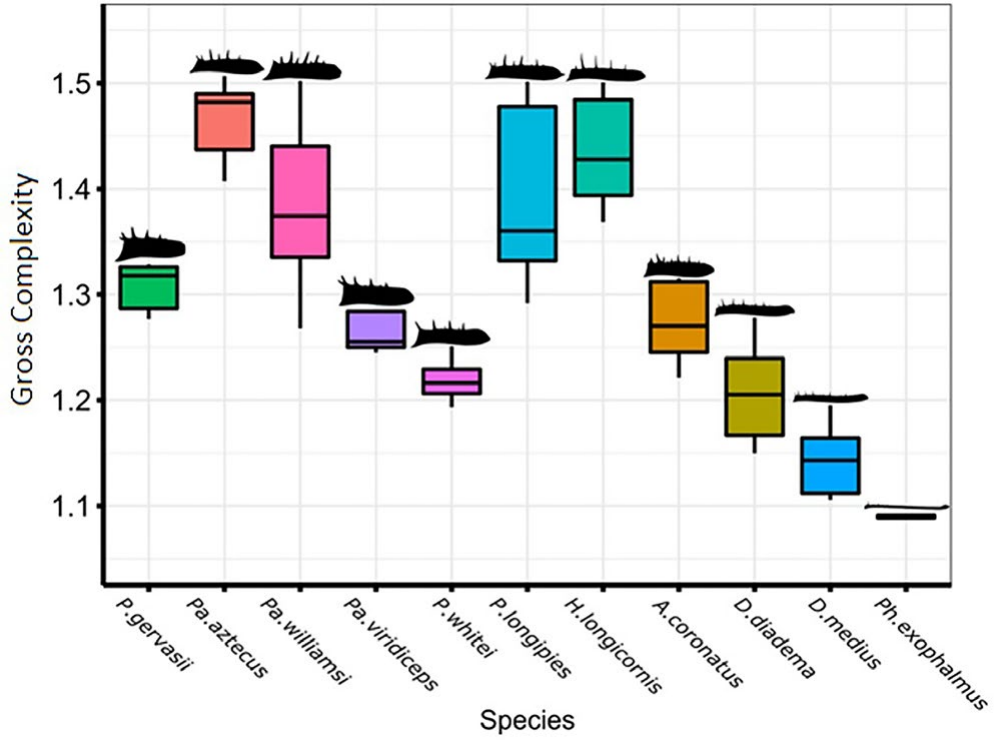
Plot of gross complexity against species in the tibia segment. Species arranged in order of absolute pedipalp length (mm): short pedipalps, left; long pedipalps, right. Notably, species with longer spines relative to the central pedipalp shaft have higher gross complexity scores. The graph also shows that gross complexity decreases with increasing pedipalp length

On a qualitative level, the main visual difference between the 4th and the 20th harmonic outlines appears to be the presence of large spines in the 20th harmonic outline. This would suggest that gross complexity is heavily influenced by these “major” spines. Indeed, initial inspection of box plots suggests that species that are characterized by larger spines relative to the central pedipalp shaft have high gross complexity scores (see Figure 2). Observationally, the total number of large spines also appears to influence gross complexity. This is best illustrated in the tibial segment, in which species with relatively long but few large spines, such as *H. longicornis* and *P. whitei*, appear to have lower gross complexity scores than other species with a larger number of “major” spines. The tibia segment was found to have statistically higher complexity (Wilcoxon, *p* = <0.001, *v* = 2,849) than the femur.

### 3.2 Species clustering

**FIGURE 3:**
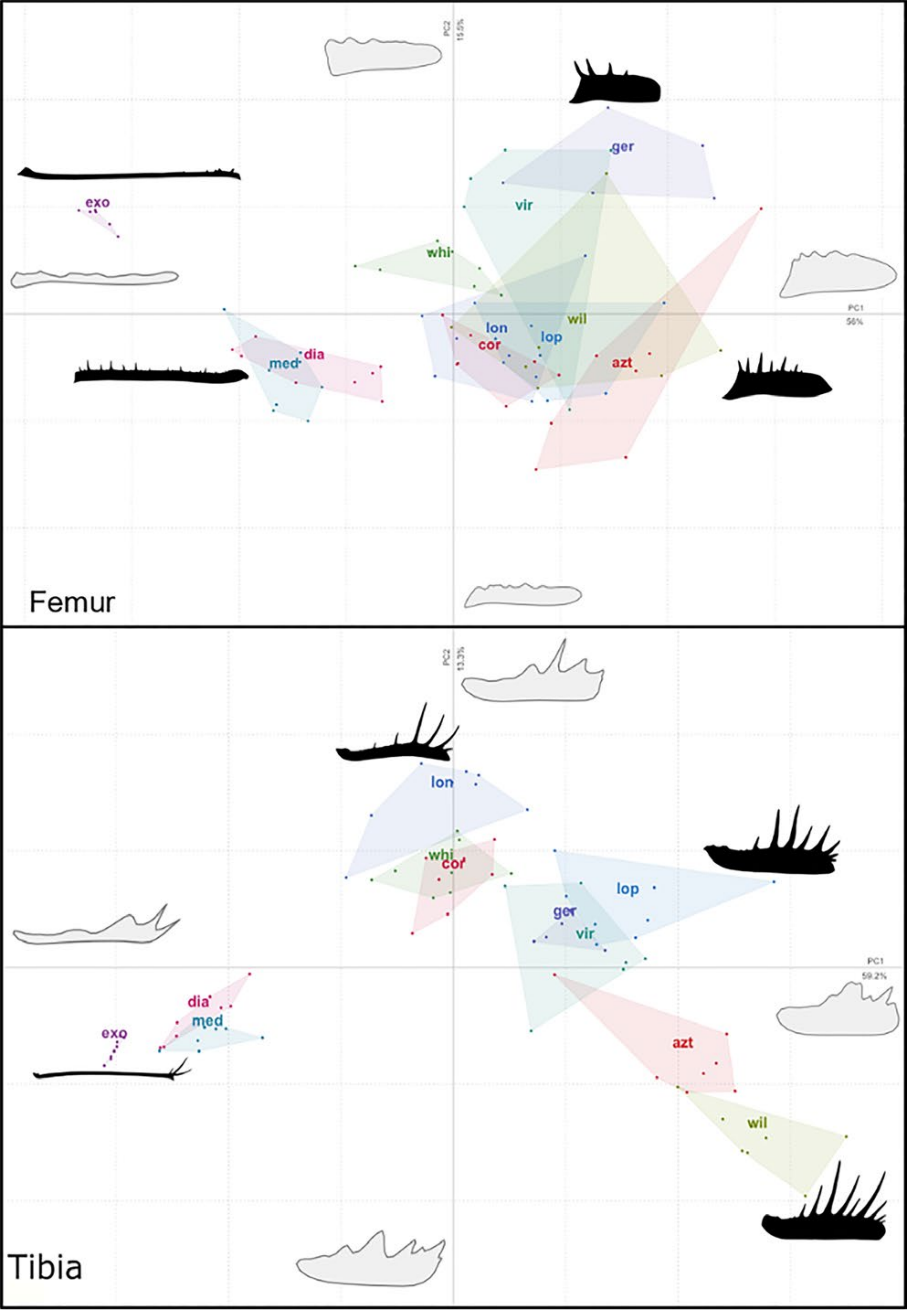
Relationship between measure of shape complexity and pedipalp length, in the tibia and femur segments. Points represent species means, and error bars show standard error around the mean

**FIGURE 4:**
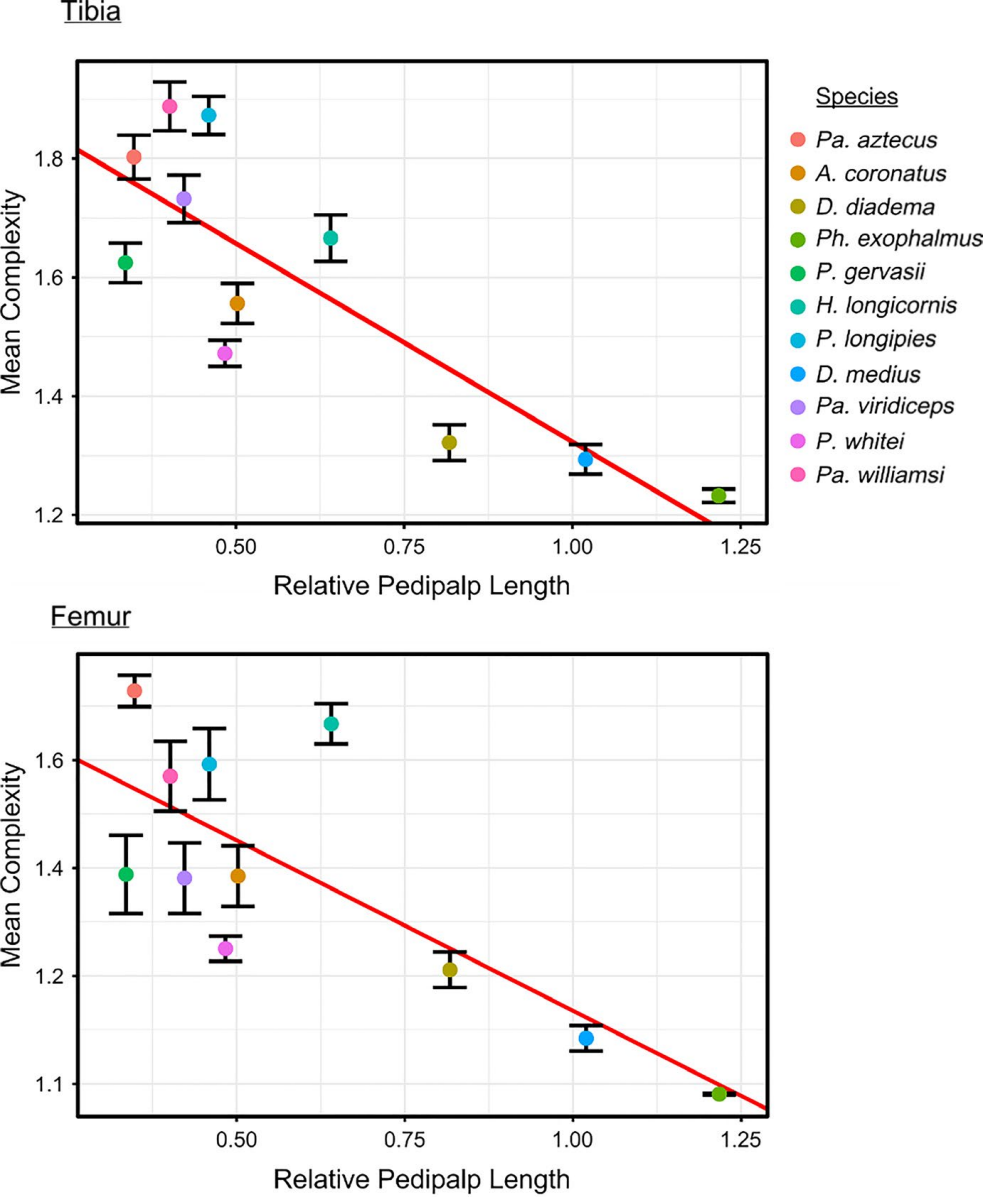
PCA of harmonic coefficients for all specimens (male and female). Black outlines visualize outlines of representative pedipalps, and gray outline is hypothetical morphs (calculated at the 20th harmonic) from extreme position on the PC axes, convex hulls represent the intraspecific variation within species. Exo = *Phrynichus exophthalmus*, dia = *Damon diadema*, med = *Damon medius*, lon = *Heterophrynus longicornis*, whi = *Phrynus whitei*, cor = *Acanthophrynus coronatus*, ger = *Phrynus barbadensis*, vir = *Paraphrynus viridiceps*, lop = *Phrynus longipes*, azt = *Paraphrynus aztecus*, wil = *Paraphrynus williamsi*

A PCA of harmonic coefficients was carried out in order to visualize intra‐ and interspecific shape variation and generate PC scores to be used in species clustering (Figure 3). The PCA appears to show a higher degree of intraspecific than interspecific differences in both segments (Figure 4). Furthermore, there was marked overlap between species in both segments, and this was particularly marked in the femur where species within the same family often overlapped. In both segments, PC1 appears to correlate to pedipalp elongation, where PC2 seems to be related to the relative position of the longest spines and the length of those spines in the tibia and femur. PC1 accounted for just over half of observed variation in both segments (femur = 56%, tibia = 59.2%) and mainly separated Phrynichidae species (*D. diadema*, *D. medius*, and *Ph. exophthalmus*) from other species. PC2 contributed roughly 15% of variation in both the femur and tibia. PC1‐10 accounted for just over 95% of total observed variation in both segments; thus, PC1‐10 were carried forward into the species clustering analysis.

Species clustering using k‐means and MANOVA methods provided markedly different results. In the femur, unsupervised k‐means clustering could only classify *Ph. exophthalmus* with 100% accuracy, placing all specimens of this species within the same cluster alongside no additional species. K‐means also placed the two *Damon* species together within a discrete genus cluster which contained no other species, but was unable to differentiate between the two species. K‐means performed poorly at correctly identifying all other species (see Table 1). In contrast, MANOVA found statistically significant differences in the pairwise comparisons between most species in the femur (see Table 2). However, like k‐means clustering, MANOVA was unable to delimitate between species within the genera *Paraphrynus* and *Damon*.

**TABLE 1:** Results of k‐means clustering applied to all specimens femoral segment

Cluster	1	2	3	4	5	6	7	8	9	10	11
*Pa. aztecus*		28.6%			14.3%	42.9%				14.3%	
*A. coronauts*			85.7%							14.3%	
*D. diadema*	100%										
*Ph. exophthalmus*											100%
*P. barbadensis*				16.7%	16.7%			16.7%	50.0%		
*H. longicornis*				50.0%	12.5%		25.0%	12.5%			
*P. longipies*			57.1%							42.9%	
*D. medius*	100%										
*Pa. viridiceps*		20.0%							80.0%		
*P. whitei*							100%				
*Pa. williamsi*		57.1%			14.3%	28.6%					

Note

K‐means tables contain the percentage of specimens that fall into each cluster. Columns represent clusters defined by k‐means, and rows represent species.

**TABLE 2:** Results of pairwise MANOVA tests between species applied to all specimens on the femoral segment

	*A. coronauts*	*D. diadema*	*Ph. exophthalmus*	*P. barbadensis*	*H. longicornis*	*P. longipies*	*D. medius*	*Pa. viridiceps*	*P. whitei*	*Pa. williamsi*
*Pa. aztecus*	<0.05	<0.001	<0.001	<0.01	<0.001	<0.01	<0.001	**N**.**S**.	<0.001	**N**.**S**.
*A. coronauts*		<0.001	<0.001	<0.001	<0.001	<0.001	<0.001	<0.01	<0.001	<0.01
*D. diadema*			<0.001	<0.001	<0.001	<0.001	**N**.**S**.	<0.001	<0.001	<0.001
*Ph. exophthalmus*				<0.001	<0.001	<0.001	<0.001	<0.001	<0.001	<0.001
*P. barbadensis*					<0.001	<0.001	<0.001	<0.05	<0.001	<0.01
*H. longicornis*						<0.001	<0.001	<0.001	<0.001	<0.001
*P. longipies*							<0.001	<0.01	<0.001	<0.05
*D. medius*								<0.001	<0.001	<0.001
*Pa. viridiceps*									<0.001	**N**.**S**.
*P. whitei*										<0.001

Note

MANOVA tables contain the magnitude p‐values for pairwise comparison, and species comparisons that show no significant differences are highlighted in bold.

K‐means performed similarly poorly in the tibial segment (Supplementary Material S2). Only *P. barbadensis* was correctly classified by k‐means, and the majority of clusters contained multiple species. MANOVA again identified many statistically significant differences in the pairwise comparison between species. However, no differences were found between *Pa. viridiceps* versus *Pa. aztecus*, and *D. diadema* versus *D. medius*.

### 3.3 Relationship between shape complexity and absolute pedipalp length

Statistically significant negative relationships between pedipalp length and size‐independent shape complexity were identified in both tibial and femoral segments (Table 3).

**TABLE 3:** Results of OLS analysis comparing pedipalp length to species mean complexity

	*a*	*b*	*R* ^2^	*F*‐statistic	*p*
Tibia	1.991	−0.668	0.667	21.03	0.001
Femur	1.484	−0.316	0.544	12.95	0.006

### 3.4 Sexual differences in shape complexity

Complexity was statistically higher in the female tibia (ANOVA, *p* = 0.037, *F* = 13.89). However, no statistically significant differences were found in the femur.

## 4 DISCUSSION

The comparative analysis presented here documents both intra‐ and interspecific differences in shape complexity within the amblypygid pedipal A principal component analysis of Fourier harmonics shows frequent overlap between species, with intraspecific shape variation often exceeding shape differences between species, even in the relatively low numbers of individuals representing each taxon in this study. MANOVA does identify statistically significant pairwise shape differences between most species. However, unsupervised k‐means struggles to define species clusters accurately. Of note, the two *Damon* species, *D. diadema* and *D. medius*, could not be separated on the basis of MANOVA, k‐means or within the PCA morphospace in either segment. Likewise, some species of *Paraphrynus* could not be differentiated on the basis of shape complexity in either segment. As such, our results do not support Hypothesis 1, that species can be statistically differentiated from one another on the basis of pedipalp outline shape.

This result has potential implications for species recognition using pedipalp characters. MANOVA, a “supervised” method which knows which specimens belong to each species a priori, could define differences between species. However, the unsupervised k‐means method, which has no prior knowledge of which species specimens belong to, could not. K‐means is arguably a better analogue for the placement of a specimen within a species as a taxonomist, like the k‐means algorithm, would have no prior knowledge of specimen's placement within a species. Notably, previous studies have identified species of *Damon* and *Paraphyrnus* partially on the basis of the relative length of spines on the femur and tibia pedipalp segments (Joya & de Armas, 2012; Prendini et al., 2005; Weygoldt, 2000). Although our results do not invalidate the use of any specific pedipalp tibia and femur characters to define species (as our analysis investigates overall pedipalp shape), we do urge caution when differentiating between closely related species using pedipalp traits. However, it should be noted that taxonomic patterns were present in the PCA, with species from the Phrynichidae family (*D. diadema, D. medius, Ph. exophthalmos*) plotting in a different part of the morphospace than the other Phrynidae species. This suggests that although gross pedipalp shape may not be a reliable way of delineating species, the shape differences may be more important at higher taxonomic levels.

In support of Hypothesis 2, a significant negative relationship between complexity and pedipalp length was identified in both segments. Visual inspection of Fourier outlines suggests that complexity predominantly reflects the relative length of major pedipalp spines (Figure 2). Interestingly, a similar pattern was revealed using GMM in *D. variegatus*, with longer pedipalps having shorter spines. Longer amblypygid pedipalps are therefore found to have relatively shorter “major” spines once normalized to centroid size. Previous work has demonstrated the importance of display in the evolution of pedipalp morphology. Roughly, 80% of conflicts in *P. longipes* are decided via display in the favor of the individual with longer pedipalps (Chapin & Reed‐Guy, 2017). Pedipalp display has also been observed in territorial contests in a number of amblypygid species (Chapin & Hill‐Lindsay, 2016; Fowler‐Finn & Hebets, 2006; Porto & Peixoto, 2013) and during the first stages of courtship across the group (Chapin & Hebets, 2016; Weygoldt, 2000). This suggests that amblypygid pedipalps are likely under the influence of sexual selection via mate choice and that resource holding potential may be correlated with pedipalp length.

It may therefore be possible that a trade‐off between pedipalp length and complexity exists, with some species taking a less complex form, with relatively shorter spines, allowing for increased pedipalp length, developing under the pressures of sexual selection and contest, to be achieved at lower developmental cost. Hypothetically, a less complex form with shorter spines may be less energetically costly to produce per unit length, which may allow individuals to achieve longer pedipalps at lower energetic cost, though as yet we know nothing of genetics of development in amblypygids, and such inferences are therefore limited.

There is some evidence for structures becoming less complex with increasing size within arachnids, although little is known about interspecific trends of shape complexity in arachnids. For example, cheliceral dentition is known to be more pronounced in juveniles and relatively decreased in the large chelicerae of adults (Solifugae), though this may in part be a function of cheliceral wear (Bird, 2015). Some pedipalp spines also appear reduced in male thelyphonids with large pedipalps (Rajashekhar & Bali, 1982). However, it is often the case that structures under the influence of sexual selection are larger and more complex in arachnids. For example, male spider legs that are used in courtship display are longer than female conspecifics and often possess elaborations such as ridges of setae (Girard & Endler, 2014; Kronestedt, 1990; Peckham & Peckham, 1889). In opilionids, the fourth legs used in male–male contest are also longer and have larger elaborated coxal apophyses not seen in females, and larger chelicerae in “major” male morphs appear to take more complex shapes than smaller “minor” morph chelicerae (Painting et al., 2015; Powell et al., 2020; da Silva Fernandes & Willemart, 2014; Willemart et al., 2009). Thus, though a trade‐off may be possible, evidence from other arachnids provides mixed support. However, it must be stressed that these examples are all intraspecific and thus may not be a good analogue for the interspecific trends we report in this work.

Another possibility is that spine length is constrained by selective regimes or developmental factors, meaning spines have simply remained at a similar absolute size across species. In this case, species with longer pedipalps would have relatively shorter spines. For example, spine length could be limited by developmental constraints relative to pedipalp length. Additionally, it is possible that absolutely longer spines may provide limited additional functional benefits to prey capture and are not important in display‐based contest, meaning there is little advantage to spine length increasing with isometry relative to pedipalp length. Pedipalp spines are thought to primarily function in prey capture, with a number of species forming pedipalp “catching baskets,” which are hypothesized to help capture and secure prey items. Little is known about amblypygid diets, but they are thought to be largely composed of primary consumer arthropods, especially insects of the orders Orthoptera and Blattodea (Chapin & Hebets, 2016). If the primary function of spines is indeed in prey capture, spine size may scale more closely with prey size than pedipalp size. Therefore, should prey size remain similar or equal across the group, spine size may also remain similar across species leading to relatively smaller spines in species that have attained longer pedipalps due to pressures of sexual selection and contest. However, this hypothesis can only be properly investigated by addressing the current paucity in data related to diet in amblypygids.

An analogue to the palpal form in Amblypygi is found in scorpion pedipalp chelae, which increase in size at rates much lower than isometry with relation to body size (Van der Meijden et al., 2010). This may be because species with vastly different body sizes feed on similarly sized prey (Polis & McCormick, 1986), suggesting there is little added benefit to larger species producing equivalently sized chelae. If a similar overlap in prey size exists across amblypygid species, spines (which are hypothetically under the most direct selection for prey capture) may also scale at rates lower than isometry relative to pedipalp length. More work is needed into amblypygid life history and social dynamics to determine the relative importance of sexual selection, selection via territorial contest, and natural selection via prey capture.

As part of this study, we have quantified sexual shape dimorphism across amblypygids for the first time. Significant sexual dimorphism in shape complexity is present in the amblypygid pedipalp tibia, with female tibiae found to be characterized by greater complexity than their male counterparts. However, we do note that the number of specimens per species in this study is low (see Supplementary Material S1). Therefore, the inferences that can be made about the presence and drivers of sexual dimorphism within individual species are limited, especially considering the high level of intraspecific shape variation observed.

The general trend of females displaying greater pedipalp shape complexity appears to be influenced by the length of the major spines, which are relatively larger in the female tibia. This may be due to the increased length of the male pedipalp, which develops under the pressure of sexual selection. A similar pattern has already been identified using GMM analysis of the pedipalps of *D. variegatus*, with the observed sexual shape dimorphism being thought to arise due to female optimizing for prey capture while males optimize for pedipalp display (McLean et al., 2020). The occurrence of this form of dimorphism in numerous species across the group may suggest that these driving forces behind sexual dimorphism in size and complexity are pervasive across the group. Interestingly, contest and courtship both involve display and are very similar in behavior across amblypygid species, suggesting this could be a common selection pressure (Weygoldt, 2000). However, more work into amblypygid life history is needed to fully understand the drivers of pedipalp morphology.

## 5 CONCLUSION

In conclusion, our results highlight the importance of considering intra‐ and interspecific variation in terms of shape, by highlighting a number of previously undocumented shape relationships across amblypygid pedipalps. Here, we find that within‐species shape complexity variation in pedipalps can occasionally exceed differences between species, and thus, caution needs to be taken when defining species on the basis of pedipalp characters. We also find that gross complexity decreases with increasing pedipalp length, potentially uncovering a trade‐off between investment in pedipalp total length (for use in sexual selection and territorial contest) and pedipalp spine length (primarily for use in prey capture). Sexual dimorphism in gross complexity is also present in the tibia and follows similar patterns seen intraspecifically in other studies, once again highlighting the pressures of display‐based contest and courtship, and trophic niche partitioning. Future studies that look to address the paucity of data on amblypygid life history, ecology, and social dynamics will be valuable in further understanding the functional morphology in this unique system.

## CONFLICT OF INTEREST

The authors declare no conflict of interest.

## AUTHOR CONTRIBUTIONS

**Callum McLean:** Conceptualization (equal); Data curation (lead); Formal analysis (lead); Funding acquisition (lead); Investigation (lead); Methodology (equal); Visualization (lead); Writing‐original draft (lead); Writing‐review & editing (equal). **Russell Garwood:** Conceptualization (equal); Methodology (supporting); Supervision (supporting); Writing‐review & editing (equal). **Charlotte Brassey:** Conceptualization (equal); Formal analysis (supporting); Investigation (supporting); Methodology (equal); Supervision (lead); Writing‐review & editing (equal).

## Supporting information

Supplementary Material

## Data Availability

Pedipalp photographs, raw binarized outlines and body, pedipalp lengths of all specimens, and code used in this study will be uploaded to the open access Figshare repository at https://doi.org/10.6084/m9.figshare.c.5289733.
