# Supplementary material for: Assessing the patterns and drivers of shape complexity in the amblypygid pedipalp
Source: Ecol Evol. 2021 Jul 14;11(15):10709–19. doi: 10.1002/ece3.7882 (PMC8328453; doi:10.1002/ece3.7882)
Supplement: Supplementary file 1 — Supplementary Material [file ECE3-11-10709-s001.docx]

**Supplementary Material 1**

| **ID** | **Museum** | ***Species*** | **Sex** | **Locality information** | **Tib** | **Fem** |
| --- | --- | --- | --- | --- | --- | --- |
| 14.viii.2001 | AMNH | *Acanthophrynus coronatus* | F | N 18 50.927 W 102 08.088 | ✓ | ✓ |
| AUGUST-6-1965 | AMNH | *Acanthophrynus coronatus* | F | Colima city, Mexico | ✓ | ✓ |
| JULY-29-1964 | AMNH | *Acanthophrynus coronatus* | F | Colima city, Mexico | ✓ | ✓ |
| NOV-24-1950 | AMNH | *Acanthophrynus coronatus* | F | Colima city, Mexico | ✓ | ✓ |
| 02-Oct-59 | AMNH | *Acanthophrynus coronatus* | M | Plan de Barrancas, Jalisco, Mexico | ✓ | ✓ |
| AUGUST-6-1966 | AMNH | *Acanthophrynus coronatus* | M | Colima city, Mexico | ✓ | ✓ |
| Nov-30-1950b | AMNH | *Acanthophrynus coronatus* | M | 18 miles S of Apatzingan, Mexico | ✓ | ✓ |
| 09/08/1967b | AMNH | *Paraphrynus aztecus* | F | 6km East of Yanga, Veracruz, Mexico | ✓ | ✓ |
| AB00109 | AMNH | *Paraphrynus aztecus* | F | Palomares, Mexico | ✓ | ✓ |
| AB00111 | AMNH | *Paraphrynus aztecus* | F | 6km East of Yanga, Veracruz, Mexico | ✓ | ✓ |
| AGG934c | AMNH | *Paraphrynus aztecus* | F | N 19.0394 W 96.8311 | ✓ | ✓ |
| 09/08/1967a | AMNH | *Paraphrynus aztecus* | M | 6km East of Yanga, Veracruz, Mexico | ✓ | ✓ |
| AB00097 | AMNH | *Paraphrynus aztecus* | M | Jardín Buena Ventura, Mexico | ✓ | ✓ |
| AGG934a | AMNH | *Paraphrynus aztecus* | M | N 19.0394 W 96.8311 | ✓ | ✓ |
| AGG934b | AMNH | *Paraphrynus aztecus* | M | N 19.0394 W 96.8312 | ✓ | ✓ |
| 8.xi.1945 #41b | AMNH | *Paraphrynus viridiceps* | F | Zaragoza, Cuba | ✓ | ✓ |
| xxx_a | AMNH | *Paraphrynus viridiceps* | F | Santa Clara, Cuba | ✓ | ✓ |
| xxx_b | AMNH | *Paraphrynus viridiceps* | F | Andros Island, Bahamas | ✓ | ✓ |
| 8.xi.1945 #41a | AMNH | *Paraphrynus viridiceps* | M | Zaragoza, Cuba | x | ✓ |
| AB00058a | AMNH | *Paraphrynus viridiceps* | M | Nassau, The Bahamas | ✓ | ✓ |
| April 13, 1953 | AMNH | *Paraphrynus viridiceps* | M | New Providence, The Bahamas | ✓ | ✓ |
| 21-24.vii.14b | AMNH | *Phrynus barbadensis* | F | 11 13'05.5" N 74 06'14.3"W | ✓ | ✓ |
| Dec-05 | AMNH | *Phrynus barbadensis* | F | Margarita Island, Venuzuela | ✓ | ✓ |
| Dec-05i | AMNH | *Phrynus barbadensis* | F | 11 0' N 63 55' W | ✓ | ✓ |
| 14.xi.1957 | AMNH | *Phrynus barbadensis* | M | Guanare, Portuguesa, Venezuela | ✓ | ✓ |
| 8.xi.1945 #41a | AMNH | *Phrynus barbadensis* | M | Zaragoza, Cuba | ✓ | ✓ |
| ARALP00262 | AMNH | *Phrynus barbadensis* | M | 09 42.150' N 82 48.99' W | ✓ | ✓ |
| JH0074 | AMNH | *Phrynus longipies* | F | Yaguate, Dominican Republic | ✓ | ✓ |
| 15.vii.2010 | AMNH | *Phrynus longipies* | F | 18 22.411'N 68 48.920'W | ✓ | ✓ |
| 11.vii.2005b | AMNH | *Phrynus longipies* | F | 18 2.035'N 66 6.012'W | ✓ | ✓ |
| 11.vii.2005a | AMNH | *Phrynus longipies* | F | 18 2.035'N 66 6.012'W | ✓ | ✓ |
| 01497ii | AMNH | *Phrynus longipies* | M | N 18 22'25.2" W 68 37'00.5" | ✓ | ✓ |
| 01497c | AMNH | *Phrynus longipies* | M | N 18 21'17.2" W 68 36'52.3" | ✓ | ✓ |
| 01497b | AMNH | *Phrynus longipies* | M | N 18 21'17.2" W 68 36'52.3" | ✓ | ✓ |
| 01497a | AMNH | *Phrynus longipies* | M | N 18 21'17.2" W 68 36'52.3" | ✓ | x |
| 9/22/2008b | AMNH | *Phrynus whitei* | F | 13.99988N 86.98917W | ✓ | ✓ |
| 11/26/2007 | AMNH | *Phrynus whitei* | F | El Viejo, Nicaragua | ✓ | ✓ |
| July-13-2006b | AMNH | *Phrynus whitei* | F | N 15 01'37.1" W89 36'57.2" | ✓ | ✓ |
| July-13-2006d | AMNH | *Phrynus whitei* | F | N 15 01'37.1" W89 36'57.2" | ✓ | ✓ |
| 01497b | AMNH | *Phrynus whitei* | M | N 18 21'17.2" W 68 36'52.3" | ✓ | ✓ |
| 01497c | AMNH | *Phrynus whitei* | M | N 18 21'17.2" W 68 36'52.3" | ✓ | ✓ |
| 01497ii | AMNH | *Phrynus whitei* | M | N 18 22'25.2" W 68 37'00.5" | ✓ | ✓ |
| 01497a | AMNH | *Phrynus whitei* | M | N 18 22'25.2" W 68 37'00.5" | ✓ | x |
| 5.Feb.1980 | AMNH | *Paraphrynus williamsi* | F | Gruta Lanquin, Alta Verapz, Guatamala | ✓ | ✓ |
| 20.AUG.1967a | AMNH | *Paraphrynus williamsi* | F | Zapaluta, Meixco | ✓ | ✓ |
| AB00005b | AMNH | *Paraphrynus williamsi* | F | Rio Selegua, Huehuetenango, Guatamala | ✓ | ✓ |
| AB00005c | AMNH | *Paraphrynus williamsi* | F | Rio Selegua, Huehuetenango, Guatamala | ✓ | x |
| 20.AUG.1967b | AMNH | *Paraphrynus williamsi* | M | Zapaluta, Meixco | ✓ | ✓ |
| AB00005a | AMNH | *Paraphrynus williamsi* | M | Rio Selegua, Huehuetenango, Guatamala | ✓ | ✓ |
| Holotype | AMNH | *Paraphrynus williamsi* | M | Zapaluta, Meixco | ✓ | ✓ |
| Paratype | AMNH | *Paraphrynus williamsi* | M | 4mi SE Zapaluta, Chiapas, Mexico | ✓ | ✓ |
| 41(A)1-9a | NMW | *Damon diadema* | F | Kenaya/Tanzania, Arc Moutains | ✓ | ✓ |
| 41(A)1-9b | NMW | *Damon diadema* | F | Kenaya/Tanzania, Arc Moutains | ✓ | ✓ |
| 41(A)1-9bc | NMW | *Damon diadema* | F | Kenaya/Tanzania, Arc Moutains | ✓ | ✓ |
| 9291b | NMW | *Damon diadema* | F | Shimoni, Kwale District, Kenya Cost Province | ✓ | ✓ |
| 41(A)1-34a | NMW | *Damon diadema* | M | Usambra Mountain Range, Tanga, Tanzania | ✓ | ✓ |
| 41(A)1-34b | NMW | *Damon diadema* | M | Usambra Mountain Range, Tanga, Tanzania | ✓ | ✓ |
| 41(A)1-34c | NMW | *Damon diadema* | M | Usambra Mountain Range, Tanga, Tanzania | ✓ | ✓ |
| 9291a | NMW | *Damon diadema* | M | Shimoni, Kwale District, Kenya Cost Province | ✓ | ✓ |
| 41(A)1-18b | NMW | *Damon medius* | F | Togo | ✓ | ✓ |
| 41(A)1-21a | NMW | *Damon medius* | F | Togo | ✓ | ✓ |
| 41(A)1-35b | NMW | *Damon medius* | F | Calabar, Nigeria | ✓ | ✓ |
| 41(A)1-35d | NMW | *Damon medius* | F | Calabar, Nigeria | ✓ | ✓ |
| 41(A)1-18a | NMW | *Damon medius* | M | Togo | ✓ | ✓ |
| 41(A)1-18c | NMW | *Damon medius* | M | Togo | ✓ | ✓ |
| 41(A)1-21e | NMW | *Damon medius* | M | Togo | ✓ | ✓ |
| 41(A)1-35a | NMW | *Damon medius* | M | Calabar, Nigeria | ✓ | ✓ |
| IV-VIII.1965 | AMNH | *Heterophrynus longicornis* | F | Caninde, Maranhao | ✓ | ✓ |
| 1897.3.4.1 | NHM | *Heterophrynus longicornis* | F | No location data | ✓ | ✓ |
| 1897.3.4.2 | NHM | *Heterophrynus longicornis* | F | No location data | ✓ | ✓ |
| Dec-23-2004b | AMNH | *Heterophrynus longicornis* | F | N 04 33' 02.4" W 52 10' 28.6" | ✓ | ✓ |
| Dec-23-2004a | AMNH | *Heterophrynus longicornis* | M | N 04 33' 02.4" W 52 10' 28.6" | ✓ | ✓ |
| 1875.7 | NHM | *Heterophrynus longicornis* | M | No location data | ✓ | ✓ |
| 1897.3.4.3 | NHM | *Heterophrynus longicornis* | M | No location data | ✓ | ✓ |
| 1904.9.13.A | NHM | *Heterophrynus longicornis* | M | No location data | ✓ | ✓ |
| 023625 | RMCA | *Phrynichus exophthalmus* | F | Kasaï, Tshienda,, Congo, D. R.S 10° 15’ ( E 024° 00’) | ✓ | ✓ |
| 023626 | RMCA | *Phrynichus exophthalmus* | F | Kasaï, Tshienda,, Congo, D. R.S 10° 15’ ( E 024° 00’) | ✓ | ✓ |
| 86089 | RMCA | *Phrynichus exophthalmus* | F | Tondeur, Congo, D. R. | ✓ | x |
| 86093 | RMCA | *Phrynichus exophthalmus* | F | Sendo, Congo, D. R. | ✓ | x |
| 66438 | RMCA | *Phrynichus exophthalmus* | M | Kasika, riv. Nzokwe, Congo, D. R.S 02° 56’ ( E 028° 32’) | ✓ | ✓ |
| 72206 | RMCA | *Phrynichus exophthalmus* | M | Ndua près Bolobo,, Congo, D. R.S 02° 10’ ( E 016° 14’) | ✓ | ✓ |
| 86082 | RMCA | *Phrynichus exophthalmus* | M | Terr. Bunia, Mt. Hoyo, grotte Yolohafiri, , Congo, D. R.N 01° 34’ ( E 030° 15’) | ✓ | ✓ |
| 86111 | RMCA | *Phrynichus exophthalmus* | M | Thysville, Congo, D. R.S 05° 15’ ( E 014° 52’) | ✓ | ✓ |

S1 – List of specimens used in this study with locality information. RMCA = Royal Museum of Central Africa, NHM = Natural History Museum, AMNH = American Museum of Natural History, NNW = Natural History Museum Vienna

**Supplementary Material 2 – Manova and K-means results for tibia segment**

| Cluster | 1 | 2 | 3 | 4 | 5 | 6 | 7 | 8 | 9 | 10 | 11 |
| --- | --- | --- | --- | --- | --- | --- | --- | --- | --- | --- | --- |
| *Pa. aztecus* |  |  |  | *42.9%* | *42.9%* | *14.3%* |  |  |  |  |  |
| *A. coronauts* |  |  |  |  |  |  | *85.7%* |  | *14.3%* |  |  |
| *D. diadema* |  | *50.0%* |  |  |  |  |  |  | *50.0%* |  |  |
| *Ph. exophthalmus* |  | *100%* |  |  |  |  |  |  |  |  |  |
| *P. barbadensis* |  |  |  |  |  |  |  | *100%* |  |  |  |
| *H. longicornis* | *62.5%* |  |  |  |  |  |  |  |  | *37.5%* |  |
| *P. longipies* |  |  | *25.0%* |  |  |  |  |  |  | *50.0%* | *25.0%* |
| *D. medius* |  | *87.5%* |  |  |  |  |  |  | *12.5%* |  |  |
| *Pa. viridiceps* |  |  |  |  |  | *33.3%* |  |  |  |  | *66.7%* |
| *P. whitei* |  |  |  |  |  |  | *100%* |  |  |  |  |
| *Pa. williamsi* |  |  |  | *75.0%* | *25.0%* |  |  |  |  |  |  |

S2a - Results of k-means clustering applied to tibial segment. K-means tables contain the percentage of specimens that fall into each cluster. Columns represent clusters defined by K-means, rows represent species.

|  | *A. coronauts* | *D. diadema* | *Ph. exophthalmus* | *P. barbadensis* | *H. longicornis* | *P. longipies* | *D. medius* | *Pa. viridiceps* | *P. whitei* | *Pa. williamsi* |
| --- | --- | --- | --- | --- | --- | --- | --- | --- | --- | --- |
| *Pa. aztecus* | *<0.001* | *<0.001* | *<0.001* | *<0.05* | *<0.001* | *<0.01* | *<0.001* | ***N.S.*** | *<0.001* | *<0.001* |
| *A. coronauts* |  | *<0.001* | *<0.001* | *<0.001* | *<0.001* | *<0.01* | *<0.001* | *<0.001* | *<0.001* | *<0.001* |
| *D. diadema* |  |  | *<0.001* | *<0.001* | *<0.001* | *<0.001* | ***N.S.*** | *<0.001* | *<0.001* | *<0.001* |
| *Ph. exophthalmus* |  |  |  | *<0.001* | *<0.001* | *<0.001* | *<0.001* | *<0.001* | *<0.001* | *<0.001* |
| *P. barbadensis* |  |  |  |  | *<0.001* | *<0.01* | *<0.001* | *<0.01* | *<0.001* | *<0.001* |
| *H. longicornis* |  |  |  |  |  | *<0.001* | *<0.001* | *<0.001* | *<0.001* | *<0.001* |
| *P. longipies* |  |  |  |  |  |  | *<0.001* | *<0.001* | *<0.001* | *<0.001* |
| *D. medius* |  |  |  |  |  |  |  | *<0.001* | *<0.001* | *<0.001* |
| *Pa. viridiceps* |  |  |  |  |  |  |  |  | *<0.01* | *<0.01* |
| *P. whitei* |  |  |  |  |  |  |  |  |  | *<0.001* |

S2b- Results of pairwise MANOVA tests between species applied to tibial segment. MANOVA tables contain the magnitude p-values for pairwise comparison, species comparisons that show no significant differences are highlighted in bold.

Supplementary Material 3 – Landmark positions for tibia and femur segment


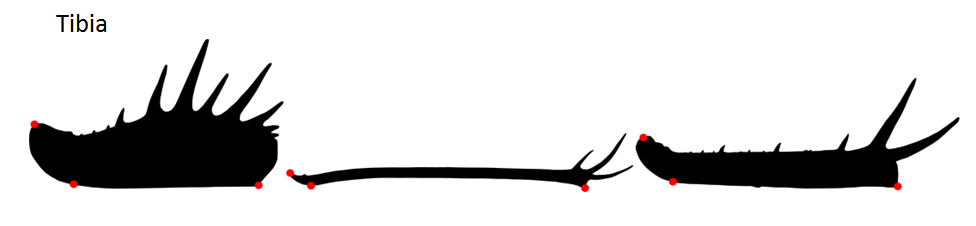


S3a – Positions of tibia landmarks. Landmarks were placed in 3 positions to aid alignment. Landmarks were placed in the following locations 1.) Articulation point between femur and tibia, 2.) Articulation point between tibia and tarsus, 3.) Inflection point at end of pedipalp central shaft. Specimen photographs were consulted to aid landmark placement where necessary.


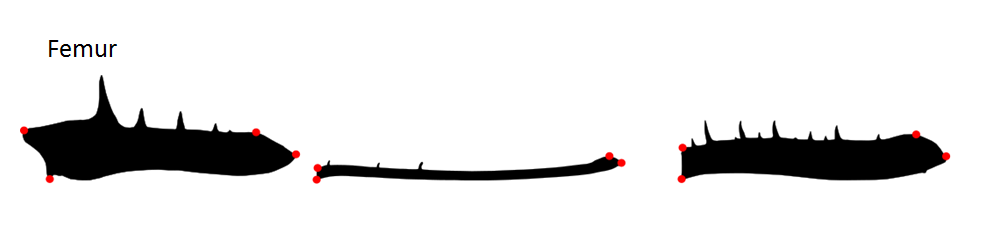


S3b – Positions of femur landmarks. Landmarks were placed in 4 positions to aid alignment. Landmarks were placed in the following locations 1.) Proximal femur end inflection point, 2.) Distal femur end inflection point, 3.) Articulation point between femur and tibia, 4.) Articulation between femur and trochanter. Specimen photographs were consulted to aid landmark placement where necessary.
